# Profuse evolutionary diversification and speciation on volcanic islands: transposon instability and amplification bursts explain the genetic paradox

**DOI:** 10.1186/s13062-016-0146-1

**Published:** 2016-09-06

**Authors:** Elysse M. Craddock

**Affiliations:** School of Natural and Social Sciences, Purchase College, State University of New York, 735 Anderson Hill Road, Purchase, NY 10577-1400 USA

**Keywords:** Speciation, Adaptive radiation, Volcanic islands, Founder effects, Genomic stress, Environmental stress, Prolonged heat stress, TE mobilization, Transposition bursts, Genome remodeling

## Abstract

**Background:**

Species-rich adaptive radiations arising from rare plant and animal colonizers are common on remote volcanic archipelagoes. However, they present a paradox. The severe genetic bottleneck of founder events and effects of inbreeding depression, coupled with the inherently stressful volcanic environment, would seem to predict reduced evolutionary potential and increased risk of extinction, rather than rapid adaptive divergence and speciation. Significantly, eukaryotic genomes harbor many families of transposable elements (TEs) that are mobilized by genome shock; these elements may be the primary drivers of genetic reorganization and speciation on volcanic islands.

**Presentation of the hypothesis:**

Here I propose that a central factor in the spectacular radiation and diversification of the endemic Hawaiian *Drosophila* and other terrestrial lineages on the Hawaiian and other oceanic islands has been repeated bursts of transposition of multiple TEs induced by the unique ecological features of volcanic habitats. Founder individuals and populations on remote volcanic islands experience significant levels of physiological and genomic stress as a consequence of both biotic and abiotic factors. This results in disruption of the usual epigenetic suppression of TEs, unleashing them to proliferate and spread, which in turn gives rise to novel genetic variation and remodels genomic regulatory circuits, facilitating rapid morphological, ecological and behavioral change, and adaptive radiation.

**Testing the hypothesis:**

To obtain empirical support for the hypothesis, test organisms should be exposed to prolonged heat stress, high levels of carbon dioxide and other volcanic gases, along with inbreeding. Data from subsequent whole genome sequencing and bioinformatics screening for TE numbers and locations would then be compared with initial pre-exposure TE information for the test strains, a labor-intensive project. Several predicted outcomes arising from the hypothesis are discussed. Currently available data are consistent with the proposed concept of stress-induced TE mobilization as a trigger of evolutionary diversification and speciation on volcanic islands.

**Implications of the hypothesis:**

The main implication is that both TEs and species should proliferate at a much higher rate on volcanic islands than elsewhere. Second, the evolvability of a lineage may correlate with the abundance and distribution of TEs in the genome. Successful colonizers of volcanic habitats with high genomic proportions of TEs may be best poised to found a speciose lineage that gives rise to a dramatic adaptive radiation. Colonizers that are depauperate in TEs are likely to be evolutionarily constrained and diversify little, if at all.

**Reviewers:**

This article was reviewed by Dr. James Shapiro and Dr. Wolfgang Miller (nominated by Editorial Board member Dr. I. King Jordan).

## Background

The terrestrial biotas of islands have featured prominently in studies of evolutionary diversification from Darwin’s time onwards [1–5]. Volcanic island archipelagoes have been of particular interest, as they harbor a disproportionately high level of biodiversity, much of which has evolved in situ from a small number of original trans-oceanic colonizers [6]. Adaptive radiations are common on remote archipelagoes such as the Hawaiian [7–13] and Galápagos [2, 14] Islands (and even on less remote archipelagoes such as the Canary Islands [15, 16]), but present a paradox. Given the depauperate genetic foundations of the many documented monophyletic lineages, the evolutionary outcomes are nothing short of astounding. For example, despite being the most isolated archipelago on earth (>3,200 km from the nearest land mass), Hawaii hosts the largest and most diverse plant clade of any oceanic island or archipelago, namely, the endemic Hawaiian lobeliads (6 genera, 126 species) derived from a single colonization event ~13 Mya [17]. Further, among Hawaiian invertebrates, there are many extraordinary endemic radiations [see 7, 10–12 for reviews]. One of the more impressive is that of the endemic Hawaiian moth genus *Hyposmocoma* which has >400 species derived from one ancestor ~15 Mya [18]. These species inhabit a vast array of ecological niches [18, 19], including aquatic zones occupied by truly amphibious caterpillars [20]. Another spectacular example is the endemic Hawaiian drosophilids, comprising an estimated 1,000 diverse species (in 2 genera), that originated from a single founder ~ 25 Mya [21, 22]. How did genomes of the founding individuals give rise to so much morphological, ecological and behavioral divergence? What genetic mechanism might have driven the explosive evolution and rapid speciation of so many plant and animal lineages in Hawaii, and in other geologically young volcanic islands such as the Galápagos?

The astonishing level of diversity in the Hawaiian drosophilid fauna was noted as early as 1913 [23], raising the question “Why are there so many species of Hawaiian *Drosophila*?” [24–26]. Decades of field and lab study of these extraordinary flies have expanded the perceived size of the fauna from early estimates of 300 species [24] to the current estimate of ~1,000 species [27] and still, a few new species continue to be discovered, even in well characterized groups such as the picture wings [28]. The question of *why* there are so many species has thus become even more pressing. Over the years, founder effects [29–32], ecological opportunity and adaptation [33–37], and behavioral shifts in the mate recognition system [38, 39], - involving processes of genetic drift, natural selection, and sexual selection, respectively - have each been emphasized as playing dominant roles in the evolution of this fauna. While certainly crucial factors in the diversification of many faunal groups in Hawaii, their complex interactions and individual causal roles in the speciation process remain ambiguous. Clearly, founder events have played a pivotal role in the proliferation of both plant and animal species in Hawaii [11, 17, 18, 29]. Further, the topographic complexity and habitat diversity of the Hawaiian Islands presented founder populations with many vacant niches to exploit. For example, availability of many endemic plant resources in the montane forests seems to have been critical in facilitating the high level of host plant specialization and speciation in the Hawaiian drosophilids [33–37], suggesting a primary mode of ecological speciation in the adaptive radiation of these flies [40]. Likewise, habitat heterogeneity and ecological opportunity have been posited as the primary driver of species diversification in many lineages of Hawaiian plants [8, 13] and invertebrates [7, 18–20, 22, 41], as well as more generally in species-rich clades in both continental and island situations [14, 42–44]. But while ecological opportunity does normally seem to be a prerequisite for adaptive radiation, it is by no means sufficient [45]. Moreover, critical questions remain about the processes that might link ecological opportunity to speciation and adaptive radiation [46]. Thus a clear-cut answer to why, and importantly, *how*, so many species have arisen in the innumerable cases of adaptive radiation on islands remains unsatisfactory. At the crux of this perplexing problem is the lack of a unifying explanation as to how the restricted gene pool of the few rare colonizers (both original founders from distant continents, and individual interisland and intraisland colonizers) could have repeatedly generated so much genetic variation so rapidly, providing the raw material for the observed evolutionary diversification.

Associated with the theory of founder effect speciation is Mayr’s concept of a genetic revolution [47] and Carson’s proposal of a genetic reorganization of polygenic balances and coadapted gene complexes [48, 49] triggered by the founder event. What this actually entails at the level of the genome has remained obscure and controversial. The several founder effect models [30, 31, 47–49] were heavily criticized by Barton and Charlesworth [50], who failed to find strong support for a major role of founder effects in speciation, concluding (in the then pre-genomic era) that “There are no empirical or theoretical grounds for supposing that rapid evolutionary divergence usually takes place in extremely small [founder] populations, ………” [see pp. 157-158 of ref. 50 for their complete conclusions]. Nonetheless, evidence from nature continues to point to a real phenomenon. What remains missing is a mechanism to explain why a founder event should trigger a genetic reorganization that apparently also expands the amount of genetic variation, thereby allowing for the production of multiple new species that are genetically distinct from the ancestral species. In this paper, I propose a central role for transposable elements, and specifically for their mobilization in founder populations in volcanic situations, in response to the heightened genomic stress they suffer during colonization of newly available habitats on a still active volcano. The idea that environmental stress may cause “genome shock,” and initiate transposon mobility and subsequent genome restructuring was pioneered by McClintock [51]. The novel aspects of the hypothesis presented here are that (1) repeated bursts of transposition are induced by the unique challenges of the dynamic volcanic environment; and (2) these transposition bursts are the key trigger of the genetic revolutions in founder populations [47–49] that subsequently lead to rapid speciation and adaptive radiation. Under this scenario, the ecological release accompanying the shift to a vacant adaptive zone (proposed in the classic evolutionary hypothesis of ecological opportunity [42, 43] to explain adaptive radiations on islands), complements the ideas presented here, playing an important sequential rather than initiating role in the process of founder effect speciation and adaptive radiation.

Transposable elements (TEs) are selfish genetic elements [52, 53] that are ubiquitous in eukaryotic genomes [54–56] and comprise the major component of the interspersed repetitive DNA. They are not restricted to euchromatin, being enriched in heterochromatic domains [57] such as centromeric, pericentromeric and telomeric regions. Their mobility and ability to replicate independently of their host genome, using either an RNA or DNA intermediate [58] make them dynamic components of the genome with the potential to have major impacts on the genomic landscape at both genic and chromosomal levels. Effects of TE mobilization can often be deleterious [54], as when gene function is disturbed or abolished by TE insertion. Thus TEs are normally kept under tight control by various small-RNA-based epigenetic mechanisms that silence TEs in the germline [59–61]. These control systems, siRNA silencing in plants and fission yeast, and the piRNA silencing pathway in animals, among others [62], appear, in fact, to have evolved as a defense against transposon proliferation in order to protect genomes from excessive damage [60, 63]. On the other hand, TE mobility can have diverse beneficial effects on genome evolution [51, 54, 64–66]. First and foremost, as a mutagenic factor, TE activity can rapidly generate genetic variation, providing abundant new raw material on which natural and sexual selection may operate. Depending on the TE and where it inserts, TE-induced mutations can have a great variety of effects from subtle to macroscopic [67, 68]. Those in regulatory regions that affect the function of promoters and enhancers are particularly important from an evolutionary point of view [69]; transcriptional networks and patterns of gene expression can undergo both major and minor modifications [70] and remarkably, there is abundant evidence for the evolutionary origin of novel regulatory sequences and networks [69–71], as well as new genes [72], from TEs. Further, ectopic recombination between TE copies can lead to chromosomal rearrangements [73] and gene (and segmental) duplications [74], remodeling genome structure.

In insects, retrotransposons that use an RNA-mediated mechanism of transposition (Class I) are more abundant [75] than DNA transposons (Class II) that use a DNA-based mechanism. Retrotransposons are also the most common type of element in plants and frequently comprise the major part of plant genomes [76]. Transposition of retrotransposons is inherently replicative, as is the case for certain categories of DNA transposons such as Helitrons [77]. Activity of these kinds of TEs thus leads to increases in TE copy numbers, and importantly, novel chromosomal locations in the genome. The accumulated TEs increase the plasticity and evolvability of their host genome [66] and set the stage for more rapid shifts in gene regulatory networks and genome restructuring that drive the process of speciation [64, 78–80]. The multiplicity of effects of mobile elements on genomes make them veritable “engines of evolution.”

Since the seminal insights of McClintock [51], there have been many reports [80–84] of the induction of TE activity by various forms of stress in a range of organisms including plants [85, 86] and the model organism *Drosophila melanogaster*, which is known to carry representatives of some 121-130 TE families [57, 87]. The stressors include both biotic factors, such as inbreeding [88, 89] and interspecific hybridization [90–92], and abiotic factors. Among environmental stressors is the physical factor of heat shock which has been demonstrated to increase transposition rates of a variety of elements by one or two orders of magnitude in diverse organisms from yeast to flies [81–83, 93, 94]. Other physical factors include cold shock, UV radiation and γ-radiation [83]. Likewise, a variety of chemicals and toxins can induce high levels of transposition, even if the exposure is very brief (*e.g*., 1.5 min exposure to ethanol vapors!) [83]. Extrinsic environmental stressors as well as a variety of cellular stresses [95] trigger bursts of transposition resulting in dramatic amplification of TE copy numbers and generation of novel mutations, some of which may be adaptive [68, 74, 96–98] and promote survival in the local changed environment. A particularly notable example is the historically high adaptive value of the melanic *carbonaria* mutant in the peppered moth *Biston betularia*, which has just recently been demonstrated to have arisen via insertion of a large, tandemly repeated TE into the first intron of the *cortex* gene [99]. It is tantalizing to consider whether the stress of coal pollution accompanying onset of the Industrial Revolution in Britain actually triggered this transposition event, estimated to have happened around 1819 [99]. Regardless, this TE-mutant, responsible for a novel phenotype, proved highly advantageous in the rapidly changing environment of the times, demonstrating the importance of transposition events for subsequent microevolution, and by extension longer-term adaptation and speciation events.

It has been proposed that mobilization of TEs in response to stress might be due to relaxation of the epigenetic control mechanisms [59]. The induced TE mobilization would lead to a transient period of genomic instability, corresponding perhaps to the stochastic phase of genetic disorganization hypothesized by Carson [48, 49] to be critical for speciation, before their activity is once more suppressed by silencing mechanisms, restoring genome stability (equivalent perhaps to the genetic reorganization phase in Carson’s hypothetical model). The situation may involve more than just relaxation of the epigenetic control mechanisms [59], with certain TEs acting as stress-responsive regulators of host gene expression [62, 68, 82, 83, 85, 100], and the altered transcriptional patterns perhaps permitting the organism to survive the stressful conditions.

## Presentation of the hypothesis

### Founder populations in volcanic habitats are subject to genomic stress

Herein, I propose that a key overlooked factor leading to explosive speciation in Hawaii and on other volcanic islands is the high level of genomic stress incurred by founder individuals colonizing newly available habitats on a recently formed island (or mountain) that is still volcanically active. I hypothesize that the volcanic context of the founder population (of flies, beetles, crickets, spiders, plants, etc.) is central to the ensuing genetic revolution and reorganization of the genome. The potential role of genomic stress, and in particular the abiotic stress of the volcanic environment, has not previously been considered in discussions of the speciation and evolution of the endemic Hawaiian *Drosophila* or any other Hawaiian faunal or floral group. The novel hypothesis outlined here posits that the genomic stress suffered by founding individuals and colonizing populations in habitats on a still active volcano induces repeated bursts of TE mobilization of a variety of normally silenced transposons. This explanation combines a crucial component of island ecology and our current understanding of evolutionary genomics of TEs to account for the accelerated evolution and adaptive radiation of endemic Hawaiian groups of terrestrial organisms.

In volcanic habitats, colonizing individuals and populations incur stress from both biotic and abiotic factors. The primary biotic or population genetic contributor to genomic stress is the extreme inbreeding associated with founder events [30] as propagules from ancestral source populations colonize each newly available island in an archipelago, or volcano within a growing island [101]. The founding propagule that survived trans-oceanic transport and arrives by chance in young volcanic terrain with suitable resources for survival and breeding could be, at an extreme, a single seed or a single fertilized female insect, carrying part of the genomes of the two parental individuals. Given the primary successional development of forests with suitable host plant resources on the volcanic lava, a single colonizing female insect with a high reproductive capacity could, in a few generations, potentially establish a founder population consisting of a hundred or so individuals. However, such a population will have lower average fitness because of inbreeding depression [102] and is expected to have significantly reduced heterozygosity as a result of allele loss, similar to that demonstrated in founding populations of invasive species [103]. Further, it is unlikely that this founder population will undergo a rapid, uninterrupted expansion to extremely high numbers (a population flush) because of the instability of volcanic habitats. Rather, because volcanic activity on a newly formed island is ongoing for thousands if not hundreds of thousands of years, each newly established population is subject to frequent fragmentation by active lava flows, along with repeated local extinctions and recolonizations of regenerated habitat within its range [104]. In addition to the stress of inbreeding [102], “hybridization” between genetically divergent population isolates previously separated by a lava barrier may cause additional genomic shock. Once dense montane forest becomes established on the barren lava, which may only require a few hundred years in tropical habitats such as Hawaii (http://hvo.wr.usgs.gov/volcanowatch/archive/1999/99_01_21.html), subpopulations may make secondary contact. The combination of different TEs and TE distributions in the inter-population hybrids may trigger another burst of TE instability and amplification during meiosis, compounding the effects of inbreeding. Not surprisingly, effects on population genetic structure resulting from the dynamic geological processes associated with young Hawaiian shield volcanoes have been documented in populations of two forest specialist species of Hawaiian *Tetragnatha* spiders isolated by an 1855 lava flow [105], and are pertinent to the potential for adaptive shifts and rapid evolution and speciation in this and other Hawaiian taxa besides the Hawaiian *Drosophila*.

Along with biotic stress, young evolving Hawaiian populations in cool montane forests also suffer several kinds of abiotic stress from their volcanic environment. One physical factor is heat stress from adjacent lava flows; molten lava has a temperature in excess of 700 °C. While populations of plants and animals in the immediate path of a lava flow and adjacent to it are rapidly incinerated, those somewhat further away experience significant thermal stress. The rapid upregulation of the heat shock genes, along with various downstream genes, in response to heat stress is a highly conserved mechanism across all organisms [106]. Even brief and quite mild thermal stress (1 h at 25 °C, compared to 16 °C control temperature) has been demonstrated to significantly change the transcriptional profile in two endemic species of Hawaiian *Drosophila* [107]. In a range of organisms from yeast to flies, heat shocks have been demonstrated to also activate transposition of a variety of elements [81–83, 94, 108]. It is pertinent that promoters of heat shock genes have been found to be particularly vulnerable to TE insertions in natural populations of *Drosophila melanogaster* [109–111], generating regulatory variation that has dramatic fitness consequences. Even more interestingly, prolonged heat stress (>1 day) in the plant *Arabidopsis thaliana* has been shown to lead to heterochromatin decondensation, reduction in nucleosome density throughout the genome, and transient transcriptional activation of several repetitive elements [112]. Assuming this is a conserved response, the open chromatin configuration of genomes of plants and insects close to volcanic lava flows would facilitate many TE insertion events, especially in the normally highly condensed heterochromatin. Further, nonhomologous recombination events between heterochromatic TEs and other repeats could lead to segmental duplications and expansion of heterochromatic domains (as well as deletions).

A geographically more widespread abiotic stress for organisms in volcanic environments derives from the chemicals in volcanic gas plumes that can be carried many miles away from the eruption site by the prevailing winds. Among the several gases released by Hawaiian volcanoes (http://volcanoes.usgs.gov/hazards/gas/index.php), the most hazardous are carbon dioxide and sulfur dioxide (a contributor to acid rain). As of now, there are no experimental data available on potential effects of these and other volcanic gases on transposition; but given the induction of transposition by very brief exposure to ethanol vapors [83], it is likely that repeated or ongoing exposures of *Drosophila* and other plant and animal populations to the chemical insult of a combination of volcanic gases will induce physiological and thereby genomic stress, particularly in meiotic cells. Along with effects of high CO_2_ levels on the insect heart [113] and central nervous system [113, 114], exposed forest insects will suffer bouts of severe hypoxia that likely will induce oxidative stress [115]. Such high levels of abiotic and cellular stress are likely to exacerbate the concurrent genomic stress of inbreeding in founder and fragmented population isolates on a volcanically active oceanic island, the synergism among the several kinds of stress producing a huge genomic shock and eliciting a correspondingly massive response.

## Bursts of transposition induced by genomic stress in volcanic environments lead to genetic reorganization and accelerated divergence and speciation

Recurring bursts of transposition increase genetic variation in founder populations, and likely accelerate the process of genomic reorganization and divergence that leads to reproductive incompatibilities and evolution of new species in volcanic habitats. They may therefore account for the striking patterns of adaptive radiation found on oceanic islands. Significantly, the small effective population size of initial founder populations and of population fragments subdivided by lava flows allows for genetic drift, and thus the fixation of newly amplified copies of TEs [116] that accumulate as a result of transpositional bursts. Even if non-adaptive [117, 118], the remodeled genomes of the colonizing populations with added copies of TE repeats in novel locations may facilitate the process of speciation. Adjacent population fragments isolated by lava flows may acquire quite different patterns of TE distribution because of independent insertion events post-separation, as well as random fixation of alternate sequences from an ancestral TE insertion polymorphism, thereby initiating genetic divergence among population isolates. Barring immigration and gene flow among isolates, these subdivided population fragments may subsequently form two or even a cluster of incipient species from the common gene pool of the previously established founder population, each with a unique genome organization distinguished by the numbers and chromosomal locations of multiple TEs and TE families. However, it is likely that formerly allopatric incipient species may come into contact and interbreed, once suitable forest habitat becomes established on the previously barren lava barrier that separated them. The juxtaposition in the F1 “hybrid” individuals of two genetically very similar genomes *except for* their varied TE distributions and abundance may trigger another burst of TE instability and amplification during meiosis, which in turn may drive another round of genome remodeling and divergence. Further exposure to abiotic stress of the volcanic environment as a subsequent lava flow extinguishes part of the evolving population during the shield-building phase of volcano growth, and also later on, accompanied by the biotic stress of yet another genetic bottleneck and round of inbreeding in surviving population isolates, may induce yet another transposition burst. In this manner, the population isolates of a species *in statu nascendi* are subject to repeated bottlenecks, repeated genomic explosions of transposition, and a very long period of genetic instability and genome remodeling during subsequent cyclical episodes of population growth and contraction, which are unquestionably critical to their rapid evolutionary divergence.

As described above, the multiple waves of stress-induced transposition bursts in genomes of new arrivals to unstable volcanic habitats, such as that in the vicinity of the still active Kilauea volcano on the Island of Hawaii (http://hvo.wr.usgs.gov/activity/kilaueastatus.php See website for daily updates), would lead to the rapid accumulation of many new mutations, along with substantial reorganization of the genomes, and altered transcriptional patterns and genome functioning of surviving plants and animals, priming them for subsequent completion of the speciation process. Transposition activity induced by the stress of volcanic environments is thus proposed as the key factor in the generation of new genetic variants, and in the genome reorganization that is the critical driver of the subsequent evolutionary changes leading to rapid speciation. TE insertions will rapidly generate a host of new mutations, some of which may be fixed by genetic drift [116], while the fates of others will be determined by the forces of natural and/or sexual selection.

Specifically, I propose that it is the insertion of TE sequences into noncoding regulatory regions of the genome, in response to volcanic stress, and the subsequent re-wiring of transcriptional networks [70] that may explain the rapid phenotypic evolution and origin of morphological novelties so common in adaptive radiations on volcanic islands. The products of such adaptive radiations frequently arise rapidly [13, 40], and exhibit much greater phenotypic variation than observed in the rest of the world for the genus or lineage [8]. Yet paradoxically, they show lower rates of structural gene evolution than expected [13, 119]. As is the case for heat-shock promoters [110, 111], regulatory regions may have a more open chromatin configuration, especially after prolonged heat stress [112]. Therefore, they may be inherently more vulnerable to TE insertions than structural gene sequences. Thus the repeated episodes of TE mobilization engendered by the dynamic volcanic setting might be predicted to cause a higher rate of regulatory sequence variation, with generation of novel genomic regulatory systems, some of which may lead to dramatically altered morphologies, ecologies, and sexual and non-sexual behaviors. Some of these evolutionary novelties may be adaptive [68, 74, 96–99], providing the basis for evolution of new species, or from time to time, a morphologically distinct clade. The Hawaiian Drosophilidae, for example, display a remarkable degree of morphological variation, with monophyletic clades characterized by bizarre modifications of mouthparts, legs, and antennae [120]. Just as TE insertions in plant genes have been shown to be associated with morphological changes [76], I posit that a major contributor to the evolution of morphological novelties in Hawaiian *Drosophila* may be TE insertions that modified the developmental program of adult body patterning, thereby initiating several novel and morphologically unique clades of flies in this remarkable radiation.

Transposition bursts of multiple transposons could also potentially lead to rewiring of sexual behaviors and production of a cluster of species differentiated only by secondary sexual characters. This phenomenon could be the driver for diversification of the clade of morphologically cryptic *Laupala* crickets endemic to Hawaiian forests [121]. Among the endemic Hawaiian *Drosophila*, courtship behaviors are extraordinarily diverse [122, 123], but species are also morphologically diverse, suggesting that sexual selection [38, 39] has acted in concert with other forces in the evolutionary diversification of these flies.

## Testing the hypothesis

### Some predicted outcomes of the hypothesis and pertinent available data

#### Species richness and rates of evolution on volcanic islands

If the dynamic volcanic environment induces recurrent transposition bursts and genome reorganization that facilitates speciation as hypothesized herein, then one would expect to observe (a) a higher level of species richness and single-island endemics in groups on volcanic islands compared to the level of diversification in the same genus on (non-volcanic) continental islands; and (b) a higher total proportion of TEs in genomes of species that evolved in situ on volcanic islands compared to those that evolved on continental islands. It may be hard to precisely test these two predictions because of the difficulty in identifying matching pairs of volcanic and non-volcanic islands of similar geological age, island area, topographic complexity, and distance from source continents (a factor that likely affects immigration rates). These are all factors that play a role in the expansion of biodiversity on islands according to the General Dynamic Model of island biogeography [124] that was developed to explain plant diversification in the Canary Islands. Evidence in support of prediction (b) has yet to be collected; this topic will be addressed later in the section *Proposed experimental studies*.

A corollary of the hypothesis of elevated levels of TE activity and genome remodeling induced by the stress of volcanic environments is the expectation for higher overall rates of evolution and speciation on volcanic islands, compared to non-volcanic islands and continents. The extremely high rates of speciation in many Hawaiian plants and animals [13, 40] are consistent with this expectation. For example, in the radiation of the Hawaiian silversword alliance, the diversification rate is much higher than in non-Hawaiian taxa [125]. Moreover, rates of regulatory gene evolution in the Hawaiian silverswords are significantly accelerated by comparison with evolutionary rates of their orthologues in the ancestral North American tarweed species [126]. For *Drosophila*, a recent careful examination of molecular diversification patterns has confirmed that there has been a significantly higher rate of diversification in the Hawaiian Drosophilidae than in other groups of the subgenus *Drosophila* [21]. Accelerated speciation rates have also been documented in the forest-dwelling Hawaiian crickets of the genus *Laupala* (38 species). In the monophyletic clade on the youngest island of Hawaii where speciation is both explosive and ongoing, the speciation rate is more than an order of magnitude greater than the average estimated rate for all arthropods [127]. These findings are consistent with that claim that TEs serve to dramatically increase rates of molecular evolution [66].

#### Rates of chromosomal rearrangement, and chromosomal length polymorphism

Along with higher rates of nucleotide sequence divergence and overall evolutionary diversification, another predicted outcome of the hypothesis is a higher rate of chromosomal rearrangement in lineages that evolved on volcanic islands due to ectopic recombination between the more numerous TE repeats. In the picture wing clade of Hawaiian *Drosophila*, Carson documented 213 paracentric inversions (40 % polymorphic) in the euchromatin of 103 species [128], generated in the course of the ~ 5.8 Myr of evolution of this monophyletic clade [40]. (This is an underestimate of the total number of inversions, since some species in the clade must be extinct [40]; moreover, rearrangements with both breakpoints in the heterochromatin, which has a much higher density of TEs, cannot be evaluated cytologically because of the underreplication of these sequences in polytene chromosomes.) On the other hand, the continental *Drosophila repleta* species group that radiated in South and North America has 296 paracentric inversions (60 % polymorphic) among 70 analyzed species [129], which appears to demonstrate a significantly higher inversion rate per species, contrary to prediction. This can likely be attributed to the much greater age (~16 Myr) of the *repleta* group [130], compared with the geological youth of the Hawaiian picture wing clade [40].

If TE insertions and chromosomal rearrangements are continuing to occur in populations on still volcanically active islands such as the young Island of Hawaii (<0.47 Myr), then one might expect to observe novel polymorphic inversions with the inverted sequence at very low frequency. Further, the newly arisen inversion(s) should be geographically restricted. The young species *Drosophila silvestris* endemic to the Island of Hawaii with its highly subdivided population structure [131] provides the chance to evaluate this prediction. Of the 11 polymorphic inversions in the species, seven are reasonably widespread [131, 132]. Four recently arisen inversions are restricted to two altitudinally and ecologically marginal populations 9 km apart, and occur at very low frequencies compared to the older inversions [133]. These two isolated demes are located on the eastern flank of Mauna Kea, a volcano that erupted vigorously throughout its history to reach ~4.5 km above sea level by the end of its shield-building stage ~130 kya and continued to erupt thereafter up until ~3,300 years ago [134]. The *D. silvestris* populations in this area are thus quite young, and belong to the derived “3-row” morphotype of the species [135]. Interestingly, in a laboratory stock of *D. silvestris,* an accidental prolonged heat stress that rendered all the adults sterile led to a novel inversion haplotype, following the bottleneck in the stock of a few surviving larvae [136]. I suggest that the heat stress activated bursts of transposition, leaving chromosome breaks from TE excisions in homologous positions between inversions *t* and *l*^*2*^ of Chromosome 4 that facilitated meiotic recombination in germline cells of a survivor to produce the novel haplotype. Although not previously recorded in this lab stock, this novel inversion haplotype was strongly selected for and rose to a frequency of 30 % in the stock after just a few generations [136].

Besides chromosome rearrangements, another chromosomal phenomenon - involving heterochromatin - is also relevant to the hypothesis. Where there have been truly massive amplification bursts of TEs in the heterochromatic domain of one chromosome of an autosomal pair, this could be microscopically detectable as a chromosomal length polymorphism; massive TE amplification bursts in the X- or Y-chromosome heterochromatin would result in sex chromosomes that are longer than usual. Clayton [137] recorded many instances of double-length autosomes and sex chromosomes among the endemic Hawaiian *Drosophila,* in both homozygous and heterozygous condition, where chromosomes have been lengthened significantly by the addition of heterochromatin. Intraspecific chromosomal length variation has been observed within individuals from time to time as well as between populations of a number of Hawaiian species [137]. I am unaware of reports of similar levels of chromosomal length polymorphism due to added heterochromatin in non-Hawaiian *Drosophila* species.

Some of the added heterochromatin in Hawaiian *Drosophila* species is comprised of highly repetitive satellite DNA sequences [138], but amounts of satellite DNA do not fully account for the additional amounts of DNA in these genomes. (For example, *Drosophila heteroneura* has a genome that is 17 Mb larger than that of its sister species *D. silvestris,* but surprisingly has ~0.8 Mb less satellite DNA than *D. silvestris* [138]). The implication is that TE amplification has also occurred within the blocks of added heterochromatin (as well as within the euchromatin). In fact, some of the satellite bands resolved on CsCl density gradients may be comprised of a mixture of TE repeats interspersed among the highly repetitive satellite repeats of the heterochromatin, rather than solely satellite sequences, given the pattern of nesting of TEs among other repetitious sequences observed in the heterochromatin of *D. melanogaster* [57].

#### Increased genome sizes

Another predicted consequence of the hypothesis proposed here is that species that evolved on volcanic islands may have larger genome sizes than related organisms that evolved on continental islands or on continents where transposition rates are more constrained. Massive amplifications of TEs induced by a variety of types of stress can add detectable amounts of DNA to the genome [97, 139], especially in heterochromatic regions, increasing genome size. In fact, genomes of 16 sampled endemic Hawaiian *Drosophila* species [138] are significantly larger (t_df=19_ = 2.57, P < 0.05, assuming unequal variances) than genomes of non-Hawaiian *Drosophila* species from continents or continental islands [140, 141]. Additionally, genome sizes among Hawaiian species are more variable than among non-Hawaiian species. This is consistent with the prediction that a substantially higher prevalence of TE repeats could increase chances of ectopic recombination, leading to duplications, and in other cases, deletions of large blocks of DNA and thus decreases in genome size.

Notably, one Hawaiian species, *D. cyrtoloma*, has the largest genome ever recorded for a *Drosophila* species [138]. Its genome, which is more than twice the size of the *D. melanogaster* genome, evolved via additions of seven heterochromatic chromosome arms. Although much of the added DNA appears to be comprised of several satellite sequences [138], it is conceivable that the amplification of satellite repeats in centromeric and pericentromeric regions in this and other Hawaiian species was triggered by transposition events. In interspecific hybrids of marsupial mammals, the dramatically extended centromeres of one parental chromosome set are due to retroelement amplification of centromeric heterochromatin in response to the biotic stress of hybridization [90]. Amplification of certain types of TEs may well cause amplification of adjacent repetitive sequences. It is known that Helitrons, for instance, are able to capture adjacent genes and genomic fragments [77]. This is significant in that it can lead to amplification of additional sequences to high copy numbers, as reported in the vesper bat *Myotis lucifugus* [142]. In this species, two *Helitron* families have undergone massive amplification to comprise at least 3 % of the genome [142]. TEs of this novel class are ubiquitous, and known to be present in *Drosophila* genomes including that of the Hawaiian species *Drosophila grimshawi* [143]. When mobilized, Helitrons replicate via a rolling circle mechanism [77, 144] which dramatically increases their amplification potential. In the genome of the non-Hawaiian *Drosophila yakuba*, there is evidence for a recent transpositional burst of the *DINE-1* Helitron [145] to generate > 5,000 copies [143] that comprise 3 % of the genome [145]. I suggest that this dynamic element could have been involved, along with other TEs, in repeated transpositional bursts and amplification of adjacent repetitive DNA in some species of Hawaiian *Drosophila*, in response to episodes of genomic stress. Given the propensity of *DINE-1* copies to accumulate in the pericentric heterochromatin [145], it is feasible that blocks of resident satellite DNA repeats could be transduced and also amplified via rolling circle replication, thereby proliferating at a higher rate than normal and compounding the effects of *DINE-1* amplification on genome size increase, whenever these Helitron sequences are mobilized. Moreover, these abundant *DINE-1* repeats, other TEs and satellite DNA sequences provide substrates for unequal crossing over that can lead to segmental duplications. These may become fixed under appropriate conditions in founder populations [146], further driving heterochromatin and genome size expansion in the course of karyotypic and genomic evolution of the endemic Hawaiian *Drosophila*.

#### Higher TE copy numbers in derived members of a lineage

A final corollary of the hypothesis that founder events and exposure to the volcanic environment trigger TE instability and proliferation is the prediction that TE copy numbers should be higher in genomes of the progressively younger members of a lineage that have dispersed down an island chain as new volcanic islands in the archipelago arose from the ocean floor and became habitable. The youngest species in such a lineage would have undergone more founder events and much more exposure to volcanic environments in the course of their evolutionary history than ancestral species on older islands that are now volcanically inactive. In accord with this prediction, Hunt and colleagues [147, 148] showed via *in situ* hybridization an inverse correlation between euchromatic copy numbers of the *Uhu* element, a *Tc-1* like transposon [149], and the age of the island to which each species is endemic in both the *planitibia* and *adiastola* species groups of the picture wing clade of Hawaiian *Drosophila*, as well as a similar relationship for the *LOA* element in the *planitibia* species group [148]. The *in situ* hybridization data also demonstrated variability in occupancy of TE euchromatic chromosomal sites among the populations and species sampled, confirming active transposition through evolutionary and geological time. Copy numbers of these particular TEs in the heterochromatin (and thus total genome copy numbers) for these species could not be precisely assessed due to underreplication of heterochromatic regions in *Drosophila* larval polytene chromosomes. However, Hunt et al*.* did note that the majority of the *in situ* labeling was concentrated at the centromeric regions of the polytene chromosomes [147], suggesting higher density and copy numbers of the *Uhu* element in the centromeric heterochromatin. Further, since TE insertions would initially be heterozygous, according to the hypothesis outlined here, one would expect high levels of TE polymorphism in natural populations currently or recently exposed to volcanic stress, such as those on younger lavas of the Island of Hawaii; this was, in fact, observed for the *Uhu* element [148].

### Proposed experimental studies

#### Methods to assay TE copy numbers and distributions

Few organisms have the giant banded polytene chromosomes of *Drosophila* species that make them so favorable for *in situ* detection of specific nucleotide sequences. Even in these species, the *in situ* technique has some limitations with respect to detection in heterochromatic regions, as mentioned above, and lacks the resolution to determine whether a positive signal at a particular chromosomal site is due to a single copy or several tandem copies of the sequence in question. Further, each target sequence must be assayed individually. For optimal detection and quantification of the TE content of an individual organism (experimental or field-collected), there is no substitute for access to the whole genome sequence (WGS). Although WGS data for most single-island endemic species of interest are currently lacking, the availability and decreasing cost of high-throughput next-generation sequencing technologies has significantly increased the feasibility of obtaining the requisite genome sequence data for analyses and comparisons of TE content. To acquire comprehensive TE data for multiple genomes requires targeted methods. In the first instance, transposon display methods [150] could be used to evaluate numbers and genome proportions of specific selected TEs. This approach could be used to compare representatives of the same genus on volcanic vs. continental islands, for example, to test the prediction of increased TE representation in species that evolved on volcanic islands, mentioned at the beginning of this section (prediction b). For a more comprehensive assessment of all TEs, several post-sequencing bioinformatics methods have been developed [151], which will facilitate the task of identifying and quantifying novel TE insertions in the genomes of treated organisms, for comparison with controls.

#### Proposed experiments

Empirical tests of the hypothesized effects of volcanic stress would require controlled exposures of organisms to the specific abiotic stressors associated with volcanic activity, followed by assays of resulting levels of TE mobilization via direct detection and quantification of new insertions in progeny of the test subjects using the methods mentioned above [150, 151]. The heat stress experiments should replicate temperatures measured in the field in unburned forest tracts adjacent to volcanic lava flows and exposures should be prolonged (for two days, or one week, for example). Effects of the various volcanic gases could be tested individually at concentrations measured in the field, as well as in a combination that replicates the prevailing volcanic gas mixture that is released. Test organisms could even be exposed *in situ* to the ambient volcanic air for specific time periods. Such experiments could be conducted within Hawaii Volcanoes National Park on the Island of Hawaii where volcanic activity is ongoing, and monitoring of volcanic activity and air quality is continuous. These experiments cannot readily use island endemic species because of the lack of WGS and baseline information on their TEs. Rather, they should initially use reference strains of model organisms with a comprehensive annotation of their TEs, such as specific isogenic strains of the fly *Drosophila melanogaster* and ecotypes of the model plant *Arabidopsis thaliana* [152, 153]. Such experiments could directly validate the proposed effects of volcanic stress on colonizing individuals by detecting transposition bursts that result in amplification and novel distributions of a variety of TEs. In fly experiments, to determine whether induced transposition rates in the germline are similar in the two sexes, progeny of both treated adult males crossed with untreated females, and treated females crossed with untreated males, should be examined. There is a common perception that spermatogenesis is more sensitive to environmental insults than oogenesis, but this probably does not apply to flies and other insects where germline stem cell divisions continue in gonads of both sexes (by contrast with the situation in mammals).

Similar experiments could be carried out to test the predicted effects of extreme inbreeding following founder events on mobilization of TE repeats. Preliminary evidence of increased TE copy numbers following founder events is available for recent colonizers of new habitats [154], as well as for strains of self-fertilizing rice recently domesticated from wild relatives [155]. Finally, the combined effects of biotic (intrinsic) and abiotic (extrinsic) stress could be empirically evaluated via an inbreeding experiment in which individuals are raised for several generations in a high CO_2_ and high temperature environment, or other combinations of stressors. As in previous explorations of changes in gene expression patterns in inbred lines of *D. melanogaster* in response to thermal stress [156], it is likely that the several stressors will interact synergistically, leading to greater transposon instability and a more profound response by the host genome than that induced by an individual stressor tested separately.

### Implications of the hypothesis

The hypothesis implies that both TEs and species should proliferate at a much higher rate on volcanic islands than elsewhere, with colonizing lineages giving rise to evolutionary innovations and adaptive radiations. Although monophyletic and speciose adaptive radiations are common outcomes on volcanic islands, curiously, some lineages fail to diversify and speciate, despite being exposed to the same volcanic stresses as organisms that undergo rapid evolutionary diversification. For example, among Hawaiian plant lineages half are monotypic consisting of a single species [157]. Among Hawaiian birds, the drepanidine honeycreeper radiation of ~50 species [158] contrasts with the restricted speciation and morphological diversity of the Hawaiian thrushes that colonized the Hawaiian archipelago about the same time as the honeycreepers [159]. The difference in realization of evolutionary potential of clades subject to the same extrinsic volcanic forces would suggest an intrinsic genetic constraint in lineages that colonize volcanic islands successfully, but then fail to diversify. One potential implication is that the diversity and proportion of TEs in the genome of a colonizer determines the evolvability of the resulting lineage. Indeed, Oliver and Greene [66] have asserted that TEs are a key factor, even a prerequisite, in the evolution of species-rich lineages. Thus evolutionarily constrained lineages may be unable to undergo the rapid genome remodeling that leads to an adaptive radiation primarily because of a severe lack of TEs in their ancestral genomes. On the other hand, lineages with abundant TEs in their genomes are equipped to respond to the stress of founder events and the harsh conditions of active volcanic habitats by generating a host of new genetic combinations as a result of bursts of TE amplification, setting the stage for profuse speciation and adaptive radiation. TEs may therefore play a critical role in the survival, rampant speciation and adaptation of plants and animals in volcanic environments, and may underlie many of the evolutionary innovations frequently associated with adaptive radiations.

## Reviewers' comments

### Reviewer's report 1: James A. Shapiro, University of Chicago, USA (nominated by Editorial Board member I. King Jordan, Georgia Institute of Technology, USA)

This theoretical paper articulates a plausible and verifiable hypothesis about the key role of transposable elements in evolutionary radiations on volcanic islands. The MS is well written and marshals much of the evidence in favor of the author’s hypothesis. Consequently, this MS merits publication as an explicitly theoretical proposal. The section detailing how the special conditions on volcanic islands can be expected to stimulate transposable element activity is particularly valuable.

Author's response: *I thank this reviewer for his positive reaction to the manuscript, and for highlighting the role of the volcanic environment in activating transposition, a central aspect of my hypothesis.*

My one reservation about the MS concerns the section on “Testing the Hypothesis.” It seems to me that proposed experimental tests should come at the end of this section, only after considering (1) the state of existing evidence relevant to the hypothesis and (2) discussing the kinds of genomic analysis on natural populations that could verify or discredit the hypothesis. I recommend that the author revise this section on “Testing the Hypothesis” so that the reader can see more clearly what the status currently is of relevant data and what the two available approaches are to gathering additional data by which to evaluate the author’s proposal. The exposition on the status of current data could benefit from a more highly organized presentation with relevant headings highlighting each set of available information.

Author's response*: The section on “Testing the Hypothesis” has been reordered and revised as recommended. Thank you for suggesting adding side headings to identify subsections on the various sources of supporting data. This, I believe, makes the text much more accessible to the reader, and is an improvement on my original presentation of this section.*

### Reviewer's report 2: Wolfgang Miller, Medical University of Vienna, Austria (nominated by Editorial Board member I. King Jordan, Georgia Institute of Technology, USA)

This hypothesis paper is a beautiful and comprehensive work linking our current knowledge of transposon biology and epigenetics with one of the most enigmatic and exciting biological questions, i.e., how species originate and diversify in isolated habitats such as remote volcanic islands. Although the species-rich fauna and flora of the Galapagos and Hawaiian islands were intensively studied over last centuries, their origin presents a paradox. The severe genetic bottleneck of founder events plus inbreeding depression, coupled with the inherently stressful volcanic environment, would seem to predict reduced evolutionary potential and increased risk of extinction, rather than rapid adaptive divergence and speciation. So how do such founder populations genetically diversify? For elaborating a comprehensive theory to solve this conceptual problem, Elysse Craddock, a well-known expert who has worked for decades on the evolution of Hawaiian Drosophila species, proposes here that dormant transposons, which were already present in the founder individuals, were reactivated by the exposure to volcanic heat and chemical stresses. Due to their innate mobility and mutability such TE bursts might have enabled the founders to modulate and reorganize their genomes and thereby to drive genetic diversity of their progeny, a prerequisite for adaptive radiation and speciation. Based on our current knowledge about TE biology and epigenetics, this theory sounds very plausible to me and hence requires experimental testing. In summary, this conceptual paper is very exciting for me to see this research direction as some kind of revival of experimental studies that were originally initiated by Paul Kammerer and colleagues one century ago at the Vivarium of Vienna, Austria, which was a famous Austrian private research institution founded by Hans Leo Przibram, Leopold von Portheim and Wilhelm Figdor. By stressing model systems like Ascidia and Amphibians by means of heat and/or light exposure the researchers had observed trans-generational phenotypic alterations. Unfortunately the researchers were unaware of the mere existence of transposons around this time. Thanks to our current knowledge of TEs and their epigenetic regulations however, it would be very exciting now to reevaluate their afterwards heavily criticized findings as recently suggested by Alexander Vargas (Vargas, A. 2009 Did Paul Kammerer discover epigenetic inheritance? A modern look at the controversial midwife toad experiments. J Exp Zool B Mol Dev Evol, 312(7):667-78. Doi: 10.1002/jez.b.21319).

Author's response: *Thank you for enthusiastically endorsing publication of this manuscript. The experimental results of the Lamarkian Paul Kammerer, long thought to be fraudulent, are indeed intriguing in the light of modern molecular knowledge of epigenetic mechanisms, as discussed in the Vargas publication. Kammerer was clearly a scientist ahead of his time. With respect to TEs, Barbara McClintock with her pioneering results and interpretations stands without peer in the history of molecular biology. Likewise, in the context of founder events and their surprising outcomes, Ernst Mayr was way ahead of his time in postulating in 1954 an accompanying “genetic revolution,” later elaborated by Hamp Carson to comprise a”phase of genetic disorganization followed by a phase of genetic reorganization.” These concepts were proposed during an era when there was minimal knowledge of the structure and organization of the genome, and well before knowledge of the prevalence and importance of transposons was mainstream among molecular biologists, let alone evolutionary biologists of the time.*

*Minor suggestions*: 1.) A highly spectacular case about host adaptability in response to environmental changes clearly triggered by TEs was just recently published in Nature (Van't Hof AE et al. 2016. The industrial melanism mutation in British peppered moths is a transposable element. Nature, 534:102-105.). I would mention this beautiful example in the text. Interestingly, in the same issue the group of Chris Jiggins reported that mutations affecting the expression of the same gene, i.e., Cortex, also affect color patterning and diversification in other insects, i.e., in butterflies of the genus *Heliconius* (Nadeau et al. 2016. The gene *cortex* controls mimicry and crypsis in butterflies and moths. Nature, 534, 106–110). In this study, however, the authors could not make a direct link between *cortex* expression patterns and TE insertions, although multiple remnants of TEs were found in the close vicinity of this gene. 2.) I am not sure that *D. melanogaster* might serve as an ideal system to test the hypothesis since after more than 100 years of intensive studies and experimental mutagenesis we never have obtained a single *de novo* species out of this system. Therefore I would suggest applying more “plastic” model systems, i.e., members of Drosophila groups that are currently under incipient speciation and therefore more”open” to respond adaptively to such experimental stresses. Inbred Hawaiian species might be prime candidates since exact analyses of TE insertion patterns before and after stress exposure via NGS and TE display methods are quite feasible in our days.

Author's response: *1.) I am most grateful to Dr. Miller for bringing this fascinating example to my attention. I was unaware of these studies, which were published after I submitted the ms. As suggested, I have included mention of this highly relevant example in the Background section where I had cited the potential adaptive value of novel TE-mutations in changing environments. 2.) Although I appreciate Dr. Miller’s comments and reasons for suggesting that D. melanogaster may not be a suitable organism for testing the hypothesis, I should point out that the proposed experiments are designed to test the proposition that genomic stress induced by volcanic environmental and founder conditions triggers bursts of TE mobilization, novel mutations and significant genome reorganization, resulting in a wide array of novel genomes among the progeny. These predicted genomic outcomes are testable in the short term, and in my view, serve as the initiating factor that makes subsequent speciation much more likely, more rapid, and more profuse than in the absence of TE amplification bursts. In essence, the stress-induced TE mobilization provides a plethora of raw material for the evolutionary process. This is the key point of my hypothesis that addresses the genetic paradox of the highly restricted genetic variability of the founder individuals. Probably hundreds of years of natural and sexual selection are needed to sift through the wide array of genomic variants produced in a founder population in a volcanic habitat before a cluster of new and uniquely adapted species is formed (the majority, perhaps, of the novel variants being evolutionary dead-ends). The longer-term outcome of explosive speciation is, of course, implicit in my hypothesis, but is not really feasible to test in an average human lifetime. No doubt, experimental validation of the hypothesis should be conducted using Hawaiian species, once data have been acquired from well characterized model organisms with rapid life cycles.*

## Abbreviations

Kya: 1,000 years ago
Mya: Million years ago
TE: Transposable element
